# Clinical and epidemiological profile of ST‐segment elevation myocardial infarction patients in a megacity of west of Iran

**DOI:** 10.1002/hsr2.1187

**Published:** 2023-05-05

**Authors:** Parisa Janjani, Sayeh Motevaseli, Yahya Salimi, Sousan Mahmoudi Bavandpouri, Arash Ziapour, Nahid Salehi, Sahar Karami

**Affiliations:** ^1^ Cardiovascular Research Center, Health Research Institute, Imam Ali Hospital Kermanshah University of Medical Sciences Kermanshah Iran; ^2^ Student Research Committee, Kermanshah University of Medical Sciences Kermanshah Iran; ^3^ Social Development & Health Promotion Research Center, Health Institute Kermanshah University of Medical Sciences Kermanshah Iran; ^4^ Department of Epidemiology, School of Health Kermanshah University of Medical Sciences Kermanshah Iran

**Keywords:** elevation myocardial infarction, epidemiology, protocol, risk factors, ST‐ segment, STEMI

## Abstract

**Background and Aims:**

Low‐ and middle‐income nations account for at least three‐quarters of cardiovascular disease deaths worldwide. This study aimed to obtain real knowledge about ST segment elevation myocardial infarction (STEMI) patients and provide the context for developing a principles for care quality improvement.

**Method:**

This cross‐sectional study was conducted from July 2018 through December 2019. The study sample consisted of1169 eligible patients based on inclusion criteria. The data were collected using the standard EROP and three specialized, trained questionnaires. The collected data were checked by the quality control officer and analyzed using Stata Version 14.

**Results:**

Patient baseline characteristics showed that body mass index, low‐density lipoprotein, high‐density lipoprotein, total cholesterol, and triglyceride levels were higher in women. Also, females recorded a considerable history of diabetes mellitus, hypertension, and hypercholesterolemia compared to men. The results also showed that most men were smokers (46.80%). Aspirin (94.27%), statins (91.48%), and clopidogrel (90.68%) were the common medications used at hospital discharge for patients.

**Conclusion:**

The present study suggests that identifying and managing modifiable risk factors can improve cardiovascular disease outcomes. Also, considering the early identification of STEMI patients with new therapies can effectively decrease the rate of cardiovascular disease and its attributed health outcomes.

## 1 INTRODUCTION

According to the World Health Organization's (WHO, 2021) reports, around 17.9 million people died from cardiovascular diseases (CVDs) in 2019, accounting for 32% of all global deaths. Ischemic heart disease (IHD) corresponds to a significant share of causes and ranks as the most prevalent among these deaths.^1^ In the interim, acute coronary syndromes (ACS) and sudden death cause most IHD‐related deaths, representing 1.8 million deaths per year.^2^, ^3^

ACS refers to patients with suspicion or confirmation of acute myocardial ischemia or infarction. ACS is classified into three traditional types, including non‐ST‐elevation myocardial infarction, ST‐elevation myocardial infarction (STEMI), and unstable angina, in which STEMI is a more common type of heart attack.^4^ A STEMI is the most severe heart attack because it obstructs one or more coronary arteries, and blocks blood flow to the heart. STEMI can result in more long‐term heart disease and increase the risk of death in patients in the short term.^5^, ^6^

Low‐ and middle‐income nations account for at least three‐quarters of CVD deaths worldwide. People in low‐ and middle‐income nations have less access to primary health care for the early detection and treatment of CVDs. Therefore, disease detection is frequently delayed in these countries, and people die from CVDs and other noncommunicable diseases at a younger age, typically during their most productive years.^7^, ^8^

In Iran, a few decades ago, the most prevalent causes of death transitioned from infectious disease to CVD. According to the Global Burden of Diseases reports (2010, 2015), CVD was the first leading cause of mortality and DALYs, so it was responsible for 46% of all deaths and 20%–23% of the burden of diseases in Iran.^9^, ^10^ According to a review of studies from the previous 40 years, IHD and stroke are the leading causes of death and DALYs in Iran.^9^ Another study predicted that the burden of CVD in Iran will rise sharply between 2005 and 2025, with CVD DALYs rising from 847,309 in Iranian adults in 2005 to 1,728,836 in 2025.^11^ The rising CVD epidemic could be linked to socioeconomic and cultural changes, an increase in smoking and dietary changes, insufficient physical activity, industrialization and urbanization, rising life expectancy, rising metabolic and physical risk factors, low access and affordability to primary care and treatment, and low compliance due to economic and psychological issues.^9^, ^10^

The results of studies show that there is an urgent need to focus on implementing existing cost‐effective policies and interventions to reduce disability and premature death due to CVD. Meanwhile, cardiovascular registries by providing real‐time data on a variety of cardiovascular disorders and allowing for the analysis of quality measures across a broad group of patients help to measure and improve the quality of care and are also useful for the development of treatment protocols, and identification of risk factors. Therefore, a registry was conducted in the heart center of megacity in the west of Iran from July 2018 to December 2019.

## 2 METHODS

### 2.1 Study design and participant recruitment

The present study area is Kermanshah Province, Iran, and according to the 2016 census, its population is 946,681 (2021 estimate: 1,047,000). Kermanshah is a largest and most central city, located in the middle of the country's western part. This study was conducted in Imam Ali Hospital, which is the first center of cardiovascular surgery and angiography, and the quality and quantity of diagnostic and therapeutic services made it the largest cardiovascular center in the west of Iran. Patients are admitted to this hospital for services such as angiocardiography, angioplasty, open heart surgery, medical management of congenital heart diseases, etc. A Registry Steering Committee, consisting of experienced cardiologists, was responsible for recruiting hospitals, monitoring the study progress in their respective regions, overlooking the overall study conduct, and approving the final study results.

This cross‐sectional study was conducted from July 2018 to December 2019. Data related to 1169 patients with myocardial infarction referred to Imam Ali Hospital from the time of admission to discharge were recorded using patient interviews and completed by further clinical and paraclinical examinations. The eligible patients based on inclusion criteria, including a definitive diagnosis of myocardial infarction with the approval of the emergency physician and in accordance with the EROP protocol, are considered the statistical population of this study. Also, the unwillingness of people to participate in research is an exclusion criterion.^12^ Ethics approval was obtained from the Ethics committee in biomedical research. Written informed consent was obtained from all participants and emphasis was placed on the confidentiality of personal information. All procedures performed in studies involving human participants were in accordance with the ethical standards of the institutional research committee and with the 1964 Helsinki Declaration and its later amendments or comparable ethical standards.

### 2.2 Measures

The data were collected by the standard EROP questionnaire and three specialized trained questionnaires. The questionnaire was administered in three stages, including: (1) upon arrival, (2) after the patient's condition stabilized, (3) during discharge and afterward by trained questioners who were stationed in the hospital (where the myocardial infarction [MI] patient was hospitalized). Interviews were conducted with the patient or his/her companion as soon as possible so that the treatment process was not disrupted. However, data collection related to anthropometric measurements, nutrition, physical activity, diagnostic‐therapeutic measures, in‐hospital accidents, and medication use was completed after the patient was transferred to the ward and his clinical condition stabilized. The results of tests, echo, and other measures in the paraclinic were extracted from the patient's file. One year after diagnosis, the subjects were referred to the hospital for re‐examination and echo and other tests.

First, the purpose of the survey was communicated to all participants and they will be assured that the data will be kept confidential.

### 2.3 Statistical analysis

The collected data were checked by the quality control officer and analyzed using Stata Version 14.

## 3 RESULTS

Table 1 summarizes the demographic characteristics of the study population. The mean (*SD*) participant's age was 60.65 (12.26) and the mean age of women 65.24 (11.42) was higher than that of men 59.43 (12.19). Most of the participants’ educational level was illiterate or in primary school, and the share of illiterate women was higher than the rest. Also, a large percentage of the study population were Kermanshah citizens.

**Table 1:** Demographic characteristics of the study population.

Variable	Total	Male	Female	*p* value
	*n*	*n* (%)	*n* (%)	
Study population	1169	923 (78.96)	246 (21.04)	
Age (*SD*); years	60.65 (12.26)	59.43 (12.19)	65.24 (11.42)	<0.001^a^
Education				
Illiterate	360 (32.17)	212 (23.98)	148 (62.98)	<0.001^b^
Primary school	275 (24.58)	225 (25.45)	50 (21.28)	
Secondary school	178 (15.91)	159 (17.99)	19 (8.09)	
Diploma	172 (15.37)	161 (18.21)	11 (4.68)	
Foghe_diplom and upper	134 (11.97)	127 (14.37)	7 (2.98)	
Residence state				
Kermanshah city	896 (76.91)	705 (76.63)	191 (77.96)	0.842^c^
Other cities	126 (10.82)	102 (11.09)	24 (9.80)	
Villages	143 (12.27)	113 (12.28)	30 (12.24)	

^a^ *T*‐test.

^b^ Fisher's exact test.

^c^ Chi‐squared test.

Patient baseline characteristics are presented in Table 2. Hemoglobin, white blood cell, platelet (PLT), creatinine, and glomerular filtration rate mean values were higher in males than females. In contrast, body mass index, low‐density lipoprotein, high‐density lipoprotein, PLT, total cholesterol, and triglycerides are higher in women.

**Table 2:** Patient baseline characteristics (mean ± *SE*).

Variable	Total	Male	Female	*p* value
		Mean ± *SD*	Mean ± *SD*	
Peak CK‐MB (IU/L)	104.71 ± 93.04	109.93 ± 95.51	85.28 ± 80.43	<0.001^a^
Hemoglobin (g/dL, gL) early	14.98 ± 1.86	15.36 ± 1.70	13.54 ± 1.70	<0.001^b^
White blood cell (WBC)	11.10 ± 3.60	11.17 ± 3.67	10.81 ± 3.32	0.164^a^
PLT	242.95 ± 73.38	233.52 ± 64.98	278.34 ± 90.49	<0.001^b^
Early creatinine	1.15 ± 0.36	1.17 ± 0.35	1.07 ± 0.39	<0.001^b^
LDL‐C (mg/dL)	98.00 ± 28.59	96.85 ± 27.29	102.43 ± 32.79	0.091^a^
HDL‐C (mg/dL)	41.03 ± 8.62	40.33 ± 8.28	43.71 ± 9.38	<0.001^b^
Total cholesterol	171.04 ± 40.05	168.18 ± 37.95	182.04 ± 45.74	<0.001^b^
Triglyceride	131.70 ± 78.62	128.28 ± 72.14	145.00 ± 98.98	0.021^a^
Body mass index (kg/m^2^)	26.36 ± 4.39	26.13 ± 4.27	27.23 ± 4.76	0.001^b^

Abbreviations: HDL‐C, high‐density lipoprotein cholesterol; LDL‐C, low‐density lipoprotein cholesterol; PLT, platelet.

^a^ Mann–Whitney *U* test.

^b^ *T*‐test.

The medical history of the study population is presented in Table 3. A large share of patients (>80%) have no medical history of MI, coronary artery bypass graft (CABG), angina, primary percutaneous coronary intervention (PPCI), or stroke. Females recorded a considerable history of diabetes mellitus (DM), hypertension, and hypercholesterolemia value compared to men. The results also showed that a significant percentage of men were smokers (46.80%). Based on Table 3, significant statistical differences were observed between men and women for a history of MI, a history of PPCI, history of angina, DM, hypertension, hypercholesterolemia, and being a current smoker.

**Table 3:** Patient medical history.

Variable	Total	Male	Female	*p* value
	*n* (%)	*n* (%)	*n* (%)	
History of MI				
Yes	125 (10.72)	109 (11.85)	16 (6.50)	0.044^a^
No	1041 (89.28)	811 (88.15)	230 (93.50)	
History of stroke				
Yes	65 (5.57)	47 (5.10)	18 (7.32)	0.177^a^
No	1103 (94.43)	875 (94.90)	228 (92.68)	
History of CABG				
Yes	38 (3.26)	31 (3.37)	7 (2.85)	0.683^b^
No	1129 (96.74)	890 (96.63)	239 (97.15)	
History of PPCI				
Yes	70 (6.00)	64 (6.96)	6 (2.44)	0.008^b^
No	1096 (94.00)	856 (93.04)	240 (97.56)	
History of Angina				
Yes	176 (15.08)	125 (13.57)	51 (20.73)	0.005^a^
No	991 (84.92)	796 (86.43)	195 (79.27)	
Diabetes mellitus				
Yes	259 (22.16)	157 (17.01)	102 (41.46)	<0.001^a^
No	910 (77.84)	766 (82.99)	144 (58.54)	
Hypertension				
Yes	534 (45.68)	353 (38.24)	181 (73.58)	<0.001^a^
No	635 (54.32)	570 (61.76)	65 (26.42)	
Hypercholesterolemia				
Yes	293 (25.11)	189 (20.52)	104 (42.28)	<0.001^a^
No	874 (74.89)	732 (79.48)	142 (57.72)	
Current smoker				
Yes	449 (38.41)	432 (46.80)	17 (6.91)	<0.001^a^
No	720 (61.59)	491 (53.20)	229 (93.09)	
Reperfusion therapy^c^				
PPCI	637 (56.07)	514 (57.37)	123 (51.25)	0.236^a^
Thrombolysis	369 (32.48)	283 (31.58)	86 (35.83)	
CABG	111 (9.50)	90 (9.75)	21 (8.54)	
No reperfusion	130 (14.44)	99 (11.05)	31 (12.92)	

Abbreviations: CABG, coronary artery bypass graft; MI, myocardial infarction; PPCI, primary percutaneous coronary intervention.

a Chi‐squared test.

b Fisher's exact test.

c Thirty‐six patients with primary PCI and 42 patients with thrombolysis finally received CABG during the index hospitalization as the CABG number overlaps with TT and PPCI.

The medications used at hospital discharge for patients are reported in Table 4. A total of 94.27% of patients received aspirin 90.68%, received clopidogrel, and 91.48% received statins. Also, significant statistical differences were observed between men and women for MRAs.

**Table 4:** Medications use at hospital discharge for patients.

Variable	Total *n* (%)	Male *n* (%)	Female *n* (%)	*p* value
At discharge	(1169)	923 (78.96%)	246 (21.04%)	
Aspirin			
Yes	1102 (94.27)	872 (94.47)	230 (93.50)	0.577^a^
No	67 (5.73)	51 (5.53)	16 (6.50)	
Clopidogrel				
Yes	1060 (90.68)	836 (90.57)	224 (91.06)	0.817^a^
No	109 (9.32)	87 (9.43)	22 (8.94)	
Prasugrel				
Yes	4 (0.34)	2 (0.22)	2 (0.81)	0.155^b^
No	1165 (99.66)	921 (99.78)	244 (99.19)	
Ticagrelor				
Yes	28 (2.40)	25 (2.71)	3 (1.22)	0.175^b^
No	1141 (97.60)	898 (97.29)	243 (98.78)	
Fondaparinux				
Yes	1 (0.09)	1 (0.11)	0 (0)	0.606^b^
No	1168 (99.91)	922 (99.89)	246 (100)	
Warfarin				
Yes	8 (0.68)	6 (0.65)	2 (0.81)	0.783^a^
No	1161 (99.32)	917 (99.35)	244 (99.19)	
Rivaroxabain				
Yes	17 (1.45%)	13 (1.41)	4 (1.63)	0.800^b^
No	1152 (98.55)	910 (98.59)	242 (98.37)	
BetaBlockers				
Yes	932 (79.73)	739 (80.07)	193 (78.46)	0.577^a^
No	237 (20.27)	184 (19.93)	53 (21.54)	
ACEinhibitors/ARBs				
Yes	853 (72.97)	676 (73.24)	177 (71.95)	0.686^a^
No	316 (27.03)	247 (26.76)	69 (28.05)	
MRAs				
Yes	133 (11.38)	114 (12.35)	19 (7.72)	0.042^a^
No	1036 (88.62)	809 (87.65)	227 (92.28)	
Digoxin				
Yes	11 (0.94)	10 (1.08)	1 (0.41)	0.328^b^
No	1158 (99.06)	913 (98.92)	245 (99.59)	
Diuretics				
Yes	163 (13.94)	124 (13.43)	39 (15.85)	0.330^a^
No	1006 (86.06)	799 (86.57)	207 (84.15)	
Ivabradine				
Yes	1 (0.09)	1 (0.11)	0 (0)	0.606^b^
No	1168 (99.91)	922 (99.89)	246 (100)	
Statins				
Yes	1063 (91.48)	842 (91.82)	221 (90.20)	0.421^a^
No	99 (8.52)	75 (8.18)	24 (9.80)	

^a^ Chi‐squared test.

^b^ Fisher's exact test.

## 4 DISCUSSION

The present study was conducted as a cardiovascular registry by providing real‐time data on STEMI in the heart center of a megacity in the west of Iran. According to the study's findings, the mean (*SD*) age of women was 65.24 (11.42), while men were 59.43 (12.19). The results of other studies that considered patients with STEMI, show that women were older than men, for example, in Lina et al.^13^ study, women were older (69.8 vs. 63.2 years; *p* < 0.001) and in the Zachura et al.^14^ study, women were also older than men (72.0 ± 11.3 vs. 64.0 ± 11.7 years; *p* < 0.0001).^13^ Other results of the present study revealed that DM, hypertension, and hypercholesterolemia were more common in women compared to men, while smoking was more common in men. A study (Lina et al. 2021) that considered the racial, ethnic, and sex disparities in patients with STEMI also reported that hypertension, and DM were more common in women, with the prevalence of hypertension being 67.9% in black women, followed by Hispanic women at 64.9%. Also, DM prevalence is reported at 58.2% in Hispanic women. Alos, women underwent fewer invasive cardiac procedures compared with men, including reperfusion, right heart catheterization, and mechanical circulatory support.^13^ The higher prevalence of some variables in women can be explained by hormonal changes, for example, affecting how their bodies use insulin, which puts them at a higher risk of developing diabetes and also blood pressure can be affected by weight gain.

The same finding was also reported in other studies.^15^, ^16^, ^17^ Furthermore, in the study that described the sex‐based differences in DM in a low‐income population in China, the prevalence of DM was 14.1% in men and 14.5% in women.

The results of the present study showed that a history of MI, CABG, angina, PPCI, stroke, and smoking was more prevalent in men than women. The results of the study are consistent with other studies that reported women were less likely to have undergone prior PCI and to have known coronary artery disease.^13^ As the effects of many risk factors can be changed, it seems management of modifiable risk factors can improve cardiovascular disease outcomes.

Based on WHO cardiovascular disease risk charts that predicted the risk in 21 global regions and involved 376,177 individuals from 85 cohorts, and 19,333 incident cardiovascular events recorded during a 10‐year follow‐up, significant variation was found across global regions in the estimated 10‐year predicted risk for a given risk factor profile. More detail of this comprehensive study highlighted the variation by an example and revealed that estimated cardiovascular disease risk ranged from 11% in Andean Latin America to 30% in central Asia for a 60‐year‐old male smoker without diabetes, with a systolic blood pressure of mm Hg and total cholesterol of 5 mmol/L. The percentage of people aged 40–64 years projected to be at greater than 20% risk ranged from less than 1% in Uganda to more than 16% in Egypt.^18^

The results showed aspirin (94.27%), statins (91.48%), and clopidogrel (90.68%) were the common medications used at hospital discharge for patients. It seems the rate of aspirin prescription is high, however, it is comparable, or higher than other studies. In many other studies also, the rate of aspirin prescription was high.^19^, ^20^ Based on the findings of studies, this might be related to the different indications of aspirin in diseases other than ACS. 22.16% of the study patients had DM, and had a history of MI which supports its high use in these diseases. Also, the high rate of Clopidogrel may be explained by lower cost of clopidogrel.^21^

The limitations of our study are as follows: (1) Some individuals with acute MI may not be able to participate in the study interview due to their health condition. So, the patient's family was asked, with the patient's approval, to give the necessary data about the patient, and if the patient's answer were necessary, the data were gathered when the patient's health state was stable. (2) Loss to follow up, to address this limitation, participants were asked to notify the researcher of any changes to their contact information or address. Throughout the recruitment phase, at least three contacts information from the participant's family was documented. Another strategy for reducing loss to follow‐up was to provide participants with free medical services as well as information about the significance of the research. Also, if a patient is unwilling to come to the hospital for the follow‐up appointments, follow‐up information will be gathered as much as possible through a home visit or a phone interview. In addition, the health information system of the hospital will also be utilized to monitor patient readmissions during the research period and the death record department will be contacted to confirm a participant's death.

## 5 CONCLUSION

STEMI is one of the most common causes of cardiac mortalities, so it is essential to understand the risk factors and clinical characteristics involved in STEMI. This study was conducted in the heart center of a megacity in the west of Iran from July 2018 to December 2019. The results of the study showed that a considerable share of myocardial infarction patients were referred to hospitals during a year in western Iran, and a significant share of those were men. The present study revealed the sex differences in the study population and that DM, hypertension, and hypercholesterolemia were more common in women. In contrast, the history of MI, CABG, angina, PPCI, stroke, and smoking was more prevalent in men than women. Other results of the present study showed that aspirin, statins, and clopidogrel were the most common medications used for hospital discharge patients.

The detailed demographics, disease symptoms, and treatment of STEMI patients obtained through this registry are crucial for assessing the efficacy of the treatments and medical services and are useful for developing future interventions. The present study suggests that identifying and managing well‐known modifiable risk factors can substantially improve cardiovascular disease outcomes. Also, considering the bold approaches to discovering new mechanisms for early identification of STEMI patients and developing new targeted therapies can effectively decrease the cardiovascular disease rate and its attributed health outcomes.

## AUTHOR CONTRIBUTIONS

**Parisa Janjani**: Conceptualization; Investigation; Writing—original draft. **Sayeh Motevaseli**: Data curation; Formal analysis. **Yahya Salimi**: Formal analysis; Methodology; Software. **Sousan Mahmoudi Bavandpouri**: Data curation. **Arash Ziapour**: Investigation; Methodology; Writing—review & editing. **Nahid Salehi**: Conceptualization; Writing—original draft. **Sahar Karami**: Conceptualization; Resources; Writing—original draft; Writing—review & editing.

## CONFLICT OF INTEREST STATEMENT

The authors declare no conflicts of interest.

## ETHICS STATEMENT

The study was approved by the Research Ethics Committee of Kermanshah University of Medical Sciences (IR.KUMS.REC.1400.252). Consent to submit has been received explicitly from all co‐authors, as well as from the responsible authorities—tacitly or explicitly—at the institute/organization where the work has been carried out, before the work is submitted.

The purpose of this research was completely explained to the participants through the cover page of the questionnaire, and they were assured that their information would be kept confidential by the researcher. Informed consent from the participants was acquired as they agreed to participate in the study by reviewing the questionnaire's cover page and clicking on the provided link. Furthermore, for participants younger than 18 years of age, the participant was asked for the consent of the parent or guardian.

## TRANSPARENCY STATEMENT

The lead author Nahid Salehi, Sahar Karami affirms that this manuscript is an honest, accurate, and transparent account of the study being reported; that no important aspects of the study have been omitted; and that any discrepancies from the study as planned (and, if relevant, registered) have been explained.

## Data Availability

Data are available upon reasonable request to the corresponding author.
