# Correction to “**Clinical and epidemiological profile of ST‐segment elevation myocardial infarction patients in a megacity of west of Iran**”

**DOI:** 10.1002/hsr2.1570

**Published:** 2023-09-17

Janjani P, Motevaseli S, Salimi Y, et al. Clinical and epidemiological profile of ST‐segment elevation myocardial infarction patients in a megacity of west of Iran. Health Sci Rep. 2023;6:e1187. (https://onlinelibrary.wiley.com/doi/full/10.1002/hsr2.1187)

In Table 1 the last variable under Education was incorrect. It should be ‘Academic degree’.

We apologize for this error.

